# Mitochondria as signaling organelles

**DOI:** 10.1186/1741-7007-12-34

**Published:** 2014-05-27

**Authors:** Navdeep S Chandel

**Affiliations:** 1 Section of Pulmonary and Critical Care Medicine, Department of Medicine, Feinberg School of Medicine, Northwestern University, Chicago, IL 60611, USA

## Abstract

Almost 20 years ago, the discovery that mitochondrial release of cytochrome c initiates a cascade that leads to cell death brought about a wholesale change in how cell biologists think of mitochondria. Formerly viewed as sites of biosynthesis and bioenergy production, these double membrane organelles could now be thought of as regulators of signal transduction. Within a few years, multiple other mitochondria-centric signaling mechanisms have been proposed, including release of reactive oxygen species and the scaffolding of signaling complexes on the outer mitochondrial membrane. It has also been shown that mitochondrial dysfunction causes induction of stress responses, bolstering the idea that mitochondria communicate their fitness to the rest of the cell. In the past decade, multiple new modes of mitochondrial signaling have been discovered. These include the release of metabolites, mitochondrial motility and dynamics, and interaction with other organelles such as endoplasmic reticulum in regulating signaling. Collectively these studies have established that mitochondria-dependent signaling has diverse physiological and pathophysiological outcomes. This review is a brief account of recent work in mitochondria-dependent signaling in the historical framework of the early studies.

## 

Hans Krebs won the Nobel Prize in Physiology or Medicine in 1953 for his discovery of the citric acid cycle. In his Nobel Lecture, Krebs stated, ‘that in some micro-organisms the cycle primarily supplies intermediates rather than energy, whilst in the animal and most other organisms it supplies both energy and intermediates’. This essentially describes the two salient functions of mitochondria described in most biochemistry textbooks: production of ATP, and the generation of intermediary metabolites. The citric acid cycle, commonly referred to as the TCA cycle, generates metabolites and reducing equivalents (NADH and FADH2). Electrons from reducing equivalents feed into the mitochondrial electron transport chain (ETC), which pumps protons across the mitochondrial inner membrane to generate an electrochemical gradient that is required both for production of ATP and for the efficient shuttling of proteins into and out of mitochondria. Mitochondrial ATP generation allows cells to maintain a high ATP/ADP ratio, which is necessary to thermodynamically drive many biochemical reactions. Mitochondria are also involved in the generation of proteins that contain heme and porphyrin moieties, a process dependent on membrane potential. The metabolites generated by the TCA cycle are precursors to the biosynthesis of many macromolecules, including lipids, carbohydrates, proteins and nucleotides. Thus, mitochondria have historically been viewed as bioenergetic and biosynthetic organelles; however, mitochondrial genetics has demonstrated that alterations in proteins that have similar effects on TCA cycle and ETC activity often yield divergent phenotypes, suggesting that mitochondrial perturbation must manifest beyond biosynthesis and bioenergetics.

In the past two decades, the reason for this has become clear, as it has been increasingly appreciated that mitochondria have developed mechanisms to communicate their biosynthetic and bioenergetics fitness to the rest of the cell and thus have signaling functions beyond their metabolic ones (Figure 1). One critical function of these is to ensure that cells do not become committed to a biological process without input on the fitness of mitochondria, risking a discrepancy between the metabolic demands of the cell and the ability of the mitochondria to meet them. In the 1990s, multiple groups pioneered the idea that mitochondria communicate with the cytosol. Their observations included (1) mitochondrial release of cytochrome c to initiate cell death [1]; (2) mitochondrial release of reactive oxygen species (ROS) to activate hypoxic gene expression [2]; (3) the localization of A-kinase-anchoring proteins (AKAPs) to the mitochondrial outer membrane, allowing cAMP-dependent protein kinase (PKA) to phosphorylate substrates on the outer mitochondrial membrane [3,4]; and (4) mitochondrial dysfunction causing induction of mitochondria-specific heat shock proteins and promoting cytosolic calcium-dependent signaling [5,6].

**Figure 1:**
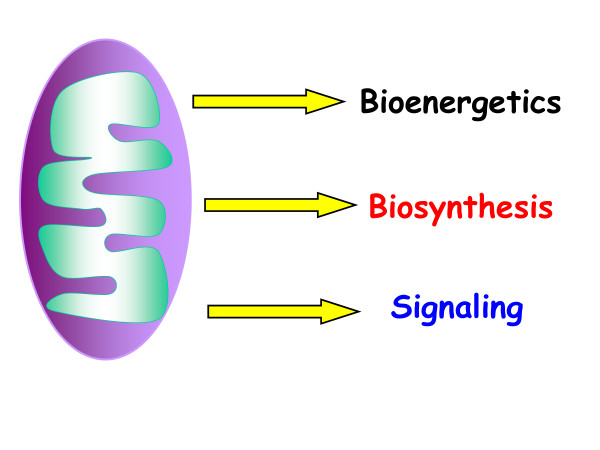
**Three essential functions of mitochondria.** Mitochondria have three distinct functions in the maintenance of homeostasis: bioenergetics, biosynthesis, and signaling.

These studies led to the emerging concept that mitochondria are signaling organelles either through the release of proteins, ROS, or metabolites, or by serving as a scaffold to configure signaling complexes (Figure 2). In the new millennium, multiple other mechanisms have been uncovered by which mitochondria communicate with the rest of the cell. Signal transduction from mitochondria to the cytosol is referred to as retrograde signaling and signal transduction from cytosol to mitochondria as anterograde signaling, and the insights of the 1990s included those emerging from studies on anterograde signaling, most notably through cytosolic calcium entering into mitochondria to regulate bioenergetics [7]. In this review, however, I will discuss only the retrograde signaling mechanisms whereby mitochondria communicate with the cell under physiological and pathological conditions.

**Figure 2:**
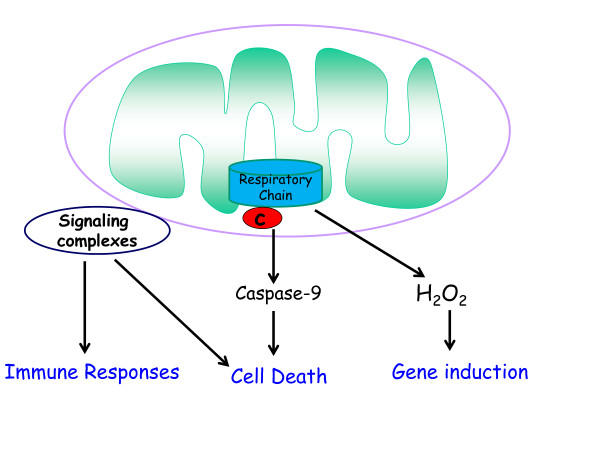
**Mitochondria regulate signaling.** Mitochondria regulate signaling pathways through release of cytochrome c to invoke caspase-dependent cell death, release of reactive oxygen species (ROS) to induce gene expression, and the use their outer membranes as a scaffold for signaling complexes, notably immune responses and control of cell death.

## Mitochondrial cytochrome c release regulates signaling

In the mid-1990s, the biology related to mitochondrial genetics was an exciting and rapidly evolving field. Mitochondria contain their own genome, which encodes a small percentage of mitochondrial proteins, with the majority encoded by nuclear DNA. How mitochondrial DNA integrates with nuclear DNA to make cells function and how miscommunication between the two types of DNA can lead to pathology continues to be a fascinating question [8]. By contrast, there was a sense that every question related to the biochemistry of mitochondria had been answered. The field’s best days were in the past. Nevertheless, I had pursued a PhD on the oxygen dependence of cytochrome c oxidase [9,10] because I thought there might be interesting biochemistry under limiting oxygen conditions (hypoxia), a condition prominent in tumors, ischemic diseases and during development *in utero*. Thus, it came as a complete surprise when in 1996 Xiaodong Wang and colleagues, using biochemical approaches, reported that cytochrome c is released from mitochondria to initiate cell death in mammalian cells [1]. Cytochrome c is an electron carrier in the mitochondria and is an essential component of the electron transport chain, and ultimately necessary for ATP generation. To me, this seemed an extraordinarily important discovery in research on mitochondria. It reenergized interest not only in the biochemical activities of mitochondria, but also in how this organelle is integrated with the rest of the cell. Soon multiple labs around the world were examining the mechanisms leading to release of cytochrome c in cells that have been instructed to die [11-13]. This led to the examination of mitochondrial ATP generation, membrane potential, and oxygen consumption in cells committed to death - all of which were things I understood. But, beyond its seminal role in cell death, the discovery of the apoptotic release of cytochrome c opened the possibility that there might be other mechanisms by which mitochondria communicate with the cell, especially under physiological conditions. It was difficult to imagine that mitochondria would play a signaling role only under conditions leading to cell death.

## Mitochondrial ROS regulate signaling

In the mid-1990s, NADPH oxidase activity had been demonstrated to promote signaling pathways involved in cell proliferation through oxidation of particular cysteine residues in proteins, modulating their activity [14-16]. By contrast, mitochondrial ROS (mROS) were proposed to be produced only under pathological conditions to invoke damage [17]. However, in the late 1990s, mROS emerged as signaling molecules that communicate between mitochondria and the rest of the cell under physiological conditions. An early example of this retrograde signaling under physiological conditions is the observation that hypoxic conditions stimulate mitochondria to release ROS, resulting in the stabilization of hypoxia inducible factors (HIFs) and the induction of genes responsible for metabolic adaptation to low oxygen [2,18]. Subsequently, mROS were shown to regulate cellular metabolism and tumor necrosis factor receptor signaling [19-21]. Eight sites in the mitochondrial inner membrane and matrix have been implicated in the production of ROS [22]. The factors that control mROS production include the concentration of oxygen available to mitochondria, the redox state of the different electron transport chain complexes and mitochondrial membrane potential [23]. In the past decade, mROS have been shown to regulate a wide range of biological processes, including oxygen sensing, epigenetics, autophagy, innate and adaptive immune responses, stem cell proliferation and differentiation, and hormone signaling [24-28].

A scientific colleague and friend once quipped, ‘If you don’t have a mechanism just say it is ROS’. There is some justification for this remark, since ROS have been linked to a wide variety of biological outcomes, including proliferation, differentiation, metabolic adaptation and senescence, though with no insight into the specific mROS targets required to invoke such diverse biological outcomes, or the mechanism involved. It is important to note that mROS targets that relay signaling could be localized in any or all of the mitochondrial matrix, the intermembrane space, or the cytosol. Furthermore, given the reactivity and toxicity of ROS at high levels, it seems likely that lower levels of mROS may be generated that invoke distinct biological outcomes. Control of different stem cell fates by ROS is an example of different levels of ROS invoking different biological outcomes. The two salient features of stem cells are their ability to self-renew, and their ability to differentiate into specialized tissues [29,30]. An emerging model is that quiescent stem cells reside at low levels of ROS and slight increases in ROS are necessary signals for self-renewal and cellular differentiation [31-33]. ROS levels above those required for self-renewal or differentiation impair these critical two stem cell properties and result in stem cell hyper-proliferation, resulting in stem cell exhaustion [34]. Going forward, it will be important to systematically quantify the ROS levels generated by mitochondria and their targets that are necessary for stem cell proliferation, differentiation and exhaustion in a given stem cell model system.

One interesting development over the past two decades has been the change in perspective on the role of mROS signaling in aging. Originally the free radical theory of aging proposed that, during the aging process, damaged mitochondria produced increasing amounts of ROS leading to tissue damage [35]. However, in most studies antioxidants have not extended lifespan of model organisms and clinical trials using antioxidants in humans have not shown any beneficial effects on age-related diseases [36]. On the contrary, recent evidence in yeast, *Caenorhabditis elegans*, and mice suggests that increasing mitochondrial generation of ROS can activate cellular stress pathways to dampen tissue degeneration, promote healthy aging and increase lifespan [37-40]. Based on the studies from the past two decades, an emerging model of mROS and signaling suggests that low levels (picomolar to nanomolar range) of mROS are necessary to maintain homeostatic biological processes, while slightly elevated levels of mROS initiate pathways for adaptation to stress. Much higher levels of mROS trigger cell death or senescence.

## Mitochondrial outer membrane as a platform for signaling

In the mid-1990s it was shown that AKAPs could be tethered to the outer mitochondrial membrane and, in subsequent years, the targeting motifs needed for AKAP binding to outer mitochondrial membrane were elucidated [4,41]. AKAPs bind to the cAMP-dependent serine/threonine kinase PKA and assemble PKA with multiple signaling proteins on a scaffold to generate a signaling hub, which can be targeted to various subcellular localizations to allow specific targeting of PKA-dependent signaling [42]. The biological significance of AKAP tethering to mitochondria was demonstrated in the late 1990s when it was shown that survival factors induce PKA-dependent phosphorylation and inactivation of BAD, a proapoptotic Bcl2 family member, specifically bound to outer mitochondrial membrane [3]. Beyond apoptosis, recent studies demonstrate that BAD tethering to mitochondria also regulates organismal glucose homeostasis [43,44]. In the past decade, mitochondria-bound PKA-AKAP assemblies have been shown to regulate oxidative metabolism, the mitochondrial fission/fusion machinery, and hypoxic responses [45,46]. This allows cellular signaling pathways to converge on the mitochondrial PKA-AKAP axis to control mitochondrial function. AKAPs have been shown to not only anchor PKA, but also act as scaffolding proteins to coordinate the activity of many other signaling enzymes such as kinases and phosphatases [47]. Thus, it is likely that there are multiple other AKAP-dependent but PKA-independent signaling pathways tethered to the outer mitochondrial membrane that remain to be discovered.

The mitochondrial outer membrane also serves as a scaffold for complexes regulating immune responses. This was first highlighted in the mid-2000s by the identification of mitochondrial antiviral signaling protein (MAVS), a crucial adaptor for RIG-I-like receptor signaling [48]. RIG-I recognizes single-stranded viral RNA and subsequently activates type I interferon production through MAVS, which is localized to the outer mitochondrial membrane [49,50]. Type-I interferons (IFNα/β) are secreted very soon after virus infection. Infected cells generate IFNβ followed shortly by the production of IFNα. Type I interferons activate interferon-stimulated genes (ISGs) as well as the NF-κB pathway. ISGs encode anti-viral proteins that produce an anti-viral state in surrounding cells in close proximity to the infected cells, to limit the spread of the virus. The activation of the NK-κB pathway leads to the production of several proinflammatory cytokines and chemokines that lead to infiltration of circulating innate cells and eventually adaptive immune T and B cells. This helps control the viral infection [51]. Recently, other innate immune molecules involved in NOD-like receptor (NLR) and TOLL-like receptor (TLR) signaling have been functionally associated with the outer mitochondrial membrane [52-54]. Exactly why these immune complexes require localization to the outer mitochondrial membrane is not fully understood [55]. Mitochondria-generated ROS are necessary for optimal function of many immune responses, and thus proximity to ROS production could be one reason why immune complexes are tethered to the outer mitochondrial membrane [56,57]. It is interesting that mitochondria are evolutionarily related to alpha-proteobacteria, and thus are descended from symbiotic pathogens. We continue to be dependent on our own pathogen - mitochondria - to mount optimal responses against foreign pathogens.

## Mitochondrial dysfunction and signaling

**Figure 3:**
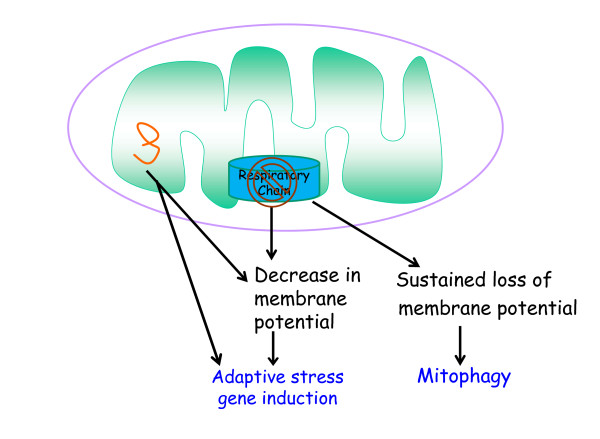
**Mitochondrial dysfunction induces stress responses or mitophagy.** Loss of mitochondrial electron transport chain function and/or misfolded proteins in mitochondrial matrix induces expression of genes through decreases in mitochondrial membrane potential. Sustained loss of mitochondrial membrane potential can invoke mitophagy, a process to eliminate dysfunctional mitochondria.

So far I have described mitochondrial signaling in the context of physiologic homeostasis. However, there are conditions where mitochondria become dysfunctional. Mitochondrial dysfunction, defined as the inability to generate ATP or metabolites, or import/export proteins, can arise when a substrate such as oxygen becomes limiting for ETC function, or through genetic alterations that lead to impairment of mitochondrial proteins. How do dysfunctional mitochondria communicate with the rest of the cell? An early clue that mitochondrial dysfunction can induce cellular responses came from the observation in the mid-1990s that the loss of mitochondrial DNA can induce nuclear encoded mitochondrial heat shock proteins (HSPs) but not cytosolic HSPs [5]. In the late 1990s, decreased mitochondrial membrane potential resulting in an increase in cytosolic calcium was one mechanism elucidated by which loss of mitochondrial DNA activates signaling pathways [6]. Subsequent studies demonstrated that accumulation of unfolded proteins within the mitochondrial matrix results in the induction of mitochondria-specific HSPs, a process referred to as the mitochondrial-unfolded response (mtUPR) [58]. The significance of the mtUPR began to be unraveled in the late 2000s by the discovery of pathways that induce the mtUPR in *C. elegans*[59,60]. Further studies demonstrated that activation of the mtUPR by mitochondrial dysfunction could increase lifespan in *C. elegans*[61,62]. Although slight or transient mitochondrial dysfunction induces adaptive stress responses, sustained mitochondrial dysfunction typically leads to elimination of mitochondria by mitophagy [63]. A given cell can contain sets of mitochondria that are functional and dysfunctional. The dysfunctional mitochondria can send signals to induce stress responses such as induction of mitochondrial HSPs until mitophagy eliminates them (Figure 3). Impairment of mitophagy can result in accumulation of dysfunctional mitochondria and disruption of calcium homeostasis, overproduction of ROS, and loss of metabolite and ATP production, all leading to the demise of the cell [64,65].

## Emerging modes of communication between mitochondria and the cell

In the past few years, other interesting modes of communication between mitochondria and the rest of the cell have been discovered. These include release of metabolites, activation of AMPK, and changes in mitochondrial dynamics. Briefly I will highlight these lively new areas. Metabolite availability has emerged as an important mechanism to control signal transduction, notably the acetylation and methylation of DNA and histones regulating epigenetics [66]. Mitochondria generate much of the acetyl-CoA and S-Adenosylmethionine (SAM) needed for protein acetylation and methylation, respectively. Thus, changes in mitochondrial biology can have profound effects on the epigenetic state [67-69]. Metabolic enzymes can also undergo acetylation at specific residues that affect their catalytic function [70]. Loss of ATP production by mitochondria (for example, during ischemia) can also regulate signaling through increasing levels of AMP and its breakdown product adenosine. The increase in AMP levels concomitant with a decrease in ATP levels activates the kinase AMPK, which halts multiple anabolic processes (metabolic demand) in the cell and promotes catabolic processes such as autophagy to ensure ample metabolic supply [71]. Adenosine can be released from cells where it engages in activation of G-protein-coupled receptors [72,73]. Thus, metabolites and adenine nucleotides typically thought of being involved in biosynthetic and bioenergetic processes are also actively involved in signaling.

The cell biology of mitochondria has rapidly evolved in the past decade, bringing about the new field of mitochondrial dynamics [74]. While the molecular details of how mitochondria move throughout the cell are still being elucidated, it is known that the ability of mitochondria to properly move around in the cell is required to properly disseminate their signals to the correct targets [75]. For example, hypoxia causes mitochondria to cluster into the perinuclear region, allowing release of ROS into the nucleus for optimal hypoxic gene expression [76]. Mitochondria in healthy cells tend to be elongated and fuse together into ‘spaghetti’ like filamentous structures. The rate of fusion and fission of mitochondria is tightly controlled [77]. Excessive fission disrupts these filamentous networks, resulting in a punctate pattern and mitochondrial dysfunction. A recent study highlights how the fission/fusion machinery integrates with cellular signaling to dictate biological outcomes. The disruption of fusion in mouse embryonic heart and in embryonic stem cells results in disruption of mouse heart development and impaired differentiation of embryonic stem cells into cardiomyocytes due to increased Ca^2+^-dependent calcineurin activity and Notch1 signaling [78].

Lastly, mitochondria tether to specific subdomains of the endoplasmic reticulum (ER) referred to as mitochondria-associated membranes or MAMs [79]. Initially, MAMs were shown to be necessary for rapid transmission of calcium (Ca^2+^) signals between the ER and mitochondria to regulate intracellular calcium levels [80]. Recent studies have demonstrated that MAMs regulate mitochondrial motility and shape, ROS and ATP production, autophagy, ER stress and immune signaling [81].

**Figure 4:**
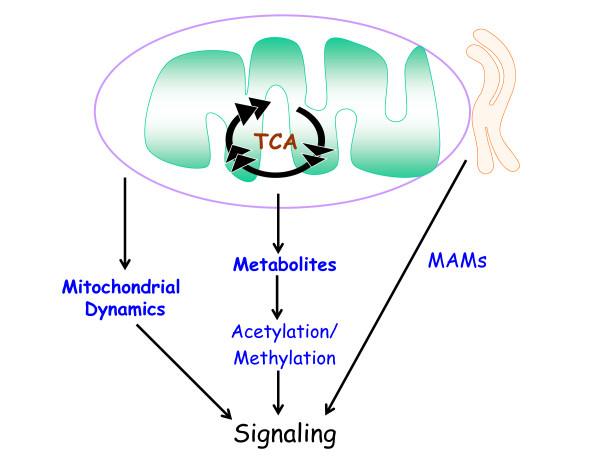
**Emerging modes of mitochondria-dependent signaling.** Mitochondrial production of metabolites and mitochondrial dynamics (motility and fission/fusion) regulate signaling. Mitochondrial interaction with specific subdomains of the endoplasmic reticulum (ER), referred to as mitochondria-associated membranes or MAMs, are emerging as important regulators of signaling transduction.

All these modes of communication are summarized in Figure 4.

## Conclusion

In the past decade, there has been an explosion of scientific inquiry in the areas of mitochondrial genetics, cell biology and biochemistry [82]. Work in these fields is increasingly converging on the control of signaling events in the rest of the cell, with important implications for regulation of physiology and common diseases. Although much has been deciphered about the types of signaling modes whereby mitochondria communicate with the rest of the cell, the molecular details remain sparse. A better understanding of the underlying mechanisms will hopefully yield insight into how to alleviate common diseases such as diabetes, cancer, and neurodegeneration, as well as provide insight into the early evolution of eukaryotes.
